# Level of surgical experience is associated with change in hip center of rotation following cementless total hip arthroplasty: A radiographic assessment

**DOI:** 10.1371/journal.pone.0178300

**Published:** 2017-05-24

**Authors:** Seung-Chan Kim, Young-Wook Lim, Soon-Yong Kwon, Woo-Lam Jo, Sung-Hun Ju, Chan-Joo Park, Choong-Woo Lee, Yong-Sik Kim

**Affiliations:** 1 Department of Orthopaedic Surgery, Seoul St. Mary’s Hospital, College of Medicine, The Catholic University of Korea, Seoul, South Korea; 2 Department of Orthopaedic Surgery, Yeouido St. Mary’s Hospital, College of Medicine, The Catholic University of Korea, Seoul, South Korea; Harvard Medical School/BIDMC, UNITED STATES

## Abstract

**Objectives:**

After total hip arthroplasty (THA), restoration of hip center of rotation (COR) is essential to ensure stability of the prosthetic hip and longevity of the prosthesis. Our aim was to determine whether, and how, the COR changed postoperatively compared to the native COR following implantation of a cementless acetabular component in anatomical position and to compare the accuracy of cup placement between two surgeons with different levels of surgical experience.

**Materials and methods:**

We evaluated 145 patients (145 hips) who underwent unilateral primary THA, who had no distorted acetabulum on the affected hip and a normal contralateral hip. Hip reconstruction was radiologically and clinically assessed at a minimum 2-year follow-up. Perioperative change in COR, initial cup position, offset, leg-length discrepancy (LLD), radiographic cup orientation, Harris Hip Score (HHS), component loosening, and dislocations were compared between the highly experienced surgeon and less-experienced surgeon groups.

**Results:**

The COR was significantly displaced in the superior and medial directions postoperatively. Significant differences were identified in the vertical COR change, initial cup position, LLD, cup inclination, and cups within safe zones, but not in the horizontal COR change, offset parameters, cup anteversion, or HHS. There were no radiographic evidence of component loosening in both groups, but three dislocations (7%) only in the group operated on by the less-experienced surgeon (*p* = 0.027).

**Conclusions:**

We found that the postoperative COR tended to be displaced in the superior and medial directions, and that the level of surgical experience strongly affected the accuracy and consistency of cup placement, particularly in COR position and cup inclination.

## Introduction

The position of the hip center of rotation (COR) following total hip arthroplasty (THA) is an essential factor that affects the joint reaction and abductor muscle forces by changing the moment arm of the abductor muscles [1]. An altered COR can be related to a discrepancy in leg length, an abnormal gait, and increased rates of acetabular and femoral component loosening [2]. Most previous studies have agreed that anatomical hip center is the optimal position for cup placement and provides the best outcome [3,4]. However, the question remains as to whether the conventional placement of cementless cups in an anatomical position can restore the original COR after THA.

The orientation of the acetabular component is another important factor that contributes to function and survival of THA [5–9]. Cup malposition following THA has been associated with an increased risk of dislocation [6,8,10–12], a limited range of motion [5], component impingement [11,13,14], increased wear of the bearing surface [6,7,15], and a greater likelihood of revision [6]. Despite advances in surgical technique and instrumentation, achievement of proper cup position still remains challenging, with potentially large variations in cup orientation and limited accuracy. Thus, improvement in the accuracy and consistency of acetabular cup placement is of great importance in minimizing the complications caused by cups placed outside the safe zones [10,12]. However, the current literature suggests that little is known regarding whether the level of surgical experience in THA leads to difference in accuracy of acetabular cup positioning in regard to COR and, if so, the nature of those differences.

In this study, we set out to determine whether, and how, the COR changed postoperatively following implantation of a cementless acetabular component in an anatomical position compared with the preoperative native COR. In addition, we aimed to determine whether a high level of surgical experience in primary THA resulted in a smaller variation in the COR position postoperatively and a lower incidence of cups placed outside the safe zones compared with a low level of surgical expertise.

## Materials and methods

Institutional Review Board (IRB) of Seoul St. Mary’s Hospital approved this study which was exempted from obtaining informed consent as the data were analyzed anonymously (approval number: KC15RISI0337). After IRB approval, 937 consecutive primary THAs (823 patients) performed at our institution between August 2009 and May 2013 were identified from our institution’s database. Of these, 145 patients (145 hips) who had undergone unilateral cementless THA for a diagnosis of osteonecrosis of the femoral head or degenerative osteoarthritis met the inclusion criteria and were considered for inclusion of this retrospective study. The inclusion criteria were a presence of a contralateral normal hip joint and a minimum follow-up period of 2 years. Hips with a distorted anatomy of the acetabulum on the affected hip—such as dysplastic hip, post-traumatic arthritis, DDH sequelae, or septic arthritis sequelae—, inflammatory arthritis and femoral neck fracture were excluded from the analysis. Patients were also excluded if they had previously undergone contralateral THA or had any abnormal anatomy on the opposite hip, as we preoperatively assessed the COR of the normal contralateral hip to obtain the native COR position of the affected hip based on our previous radiographic analysis demonstrating no significant bilateral variation in hip joint geometry, especially in the COR position [16].

All 145 THAs were performed in a tertiary hospital by two surgeons with different levels of surgical experience of, and expertise in, THA. The patients were divided into two groups based on which surgeon performed the operation: the test group consisted of 101 patients operated on by the highly experienced surgeon (the senior author), and the control group consisted of 44 patients operated on by the less-experienced surgeon (one of the authors). At the time the first included operation was carried out, the highly experienced surgeon had already performed over 1000 THAs (more than fifteen years’ experience of practice), while the less-experienced surgeon had performed fewer than 20 (less than one year’s experience of practice) and the latter performed less than 150 THAs by completion of the study. During the study period, the highly experienced surgeon performed approximately 200 THAs per year and the less-experienced surgeon performed 40 THAs per year on average.

In total, 76 females and 69 males with a mean age of 52 years (range, 20 to 86) were included in the study. The patient demographics—including the number of patients, age, gender distribution, body mass index, acetabular cup size, head diameter, and preoperative diagnosis—are shown in Table 1. There were no significant differences between the two groups in any of the variables.

**Table 1:** Patient demographics and clinical data.

Data	Highly experienced surgeon	Less-experienced surgeon	*p*-value
Number of patients (hips)	101 (101)	44 (44)	
Age (years; range)	50.9 (20 to 82)	55.7 (29 to 86)	0.087^#^
Gender (Female: Male)	54: 47	22: 22	0.701^†^
BMI (kg/m^2^; range)	24.0 (16.2 to 37.9)	23.6 (18.7 to 34.5)	0.560^#^
Diagnosis (n; %)			0.929^†^
ONFH	89 (88)	39 (89)	
Osteoarthritis	12 (12)	5 (11)	
Cup size (mm; range)	54.0 (48 to 60)	54.5 (50 to 64)	0.786^‡^
Ceramic head (n; %)			0.858^†^
32 mm	22 (22)	9 (20)	
36 mm	79 (78)	35 (80)	

# Independent *t*-test

† Chi-square test

‡ Mann-Whitney test

BMI = body mass index

ONFH = osteonecrosis of the femoral head

All of the 145 THAs were equally performed with the patient in the lateral position using a posterolateral approach with a short external rotator preservation procedure to enhance joint stability [17]. After obtaining an exposure of the acetabular rim around its entire circumference, the acetabulum was reamed with subsequent 2-mm increments until all cartilage was removed. Then, we aimed to place the cup in a press-fit manner at the anatomical COR referencing the acetabular rim following the removal of marginal osteophytes and the true acetabular floor as landmarks. The two surgeons both set the goal of cup orientation as 40° of inclination and 15° of anteversion, and positioned the cup using a mechanical guide. With ceramic-on-ceramic bearings (BIOLOX^®^ Delta; CeramTec AG, Polchingen, Germany), a cementless hemispheric porous-coated acetabular component (BENCOX^®^; Corentec, Cheonan, Korea) and a cementless double-tapered wedge femoral component with a fixed neck-shaft angle of 135° (BENCOX^®^; Corentec) were used in all hips. Patients were instructed to begin walking on the first or second postoperative day with the assistance of a frame or two crutches.

**Fig 1:**
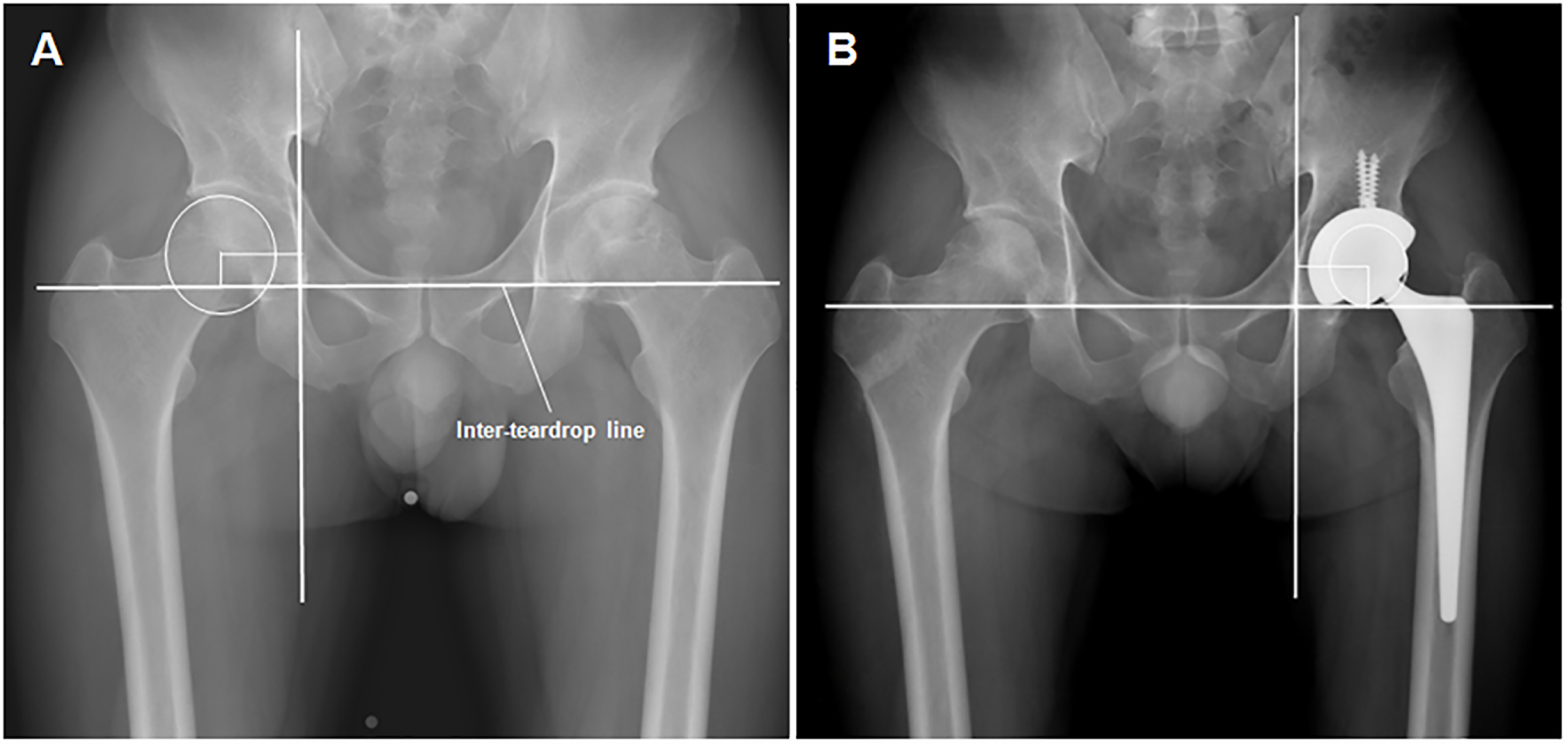
Pre- and postoperative anteroposterior pelvic radiographs of a 46-year-old male patient with osteonecrosis of the femoral head. (A) Preoperative anteroposterior template radiograph of the pelvis and (B) postoperative anteroposterior radiograph in a 46-year-old male patient with osteonecrosis of the femoral head show the radiographic measurements of the hip center of rotation (COR) and cup orientation. The inter-teardrop line is drawn between the floors of the acetabular teardrops. The actual measurements for determining the COR position are made on the unaffected side of the hip using a 10-cm standard calibration marker preoperatively (A), and on the reconstructed hip using the implanted prosthetic head postoperatively (B). The vertical height of the COR is measured as the distance between the (A) pre- or (B) postoperative COR and the inter-teardrop line. The horizontal distance of the COR is measured as the distance from the (A) pre- or (B) postoperative COR to the floor of the acetabular teardrop. The angle of inclination is the angle between the long axis of the opening ellipse and the inter-teardrop line. The angle of anteversion is calculated using an inverse sinus function (= arcsin (short axis/long axis)).

In all patients, preoperative planning with digital templating on standardized radiographs of the Picture Archiving and Communication System (PACS; Marosis, Infinite, Seoul, Korea) was performed. The radiographs comprised an anteroposterior view of the pelvis centered over the pubic symphysis with the hips at 15° of internal rotation and lateral views of both hips [18]. On the contralateral hip with normal hip joint geometry and, thus, a native COR, the center of femoral head was confirmed using a concentric circular region of interest that was digitally drawn to best fit the femoral head, and the preoperative vertical height and horizontal distance of the COR were determined (Fig 1A). The vertical height of the COR was defined as the distance between the pre- or postoperative COR and the inter-teardrop line, and the horizontal distance of the COR (i.e. acetabular offset preoperatively or cup offset postoperatively) as the distance from the pre- or postoperative COR to the floor of the acetabular tear drop (Fig 1A and 1B) [16,19]. Also, femoral offset and hip offset (a combination of acetabular and femoral offset) were measured preoperatively as described in our previous study [16]. Each template radiograph included a standard 10-cm calibration marker on the unaffected hip as close to the coronal plane of the hip joint as possible, facilitating adjustment of the magnification. Thus, we were able to make the actual measurements of the vertical height and horizontal distance of the COR and the offset preoperatively.

Routine follow-up visits were scheduled for 6 weeks, 3, 6, 12 months, and yearly thereafter. Clinical evaluation was performed using the Harris Hip Score (HHS). The COR position, femoral offset, hip offset, leg-length discrepancy (LLD), cup inclination, and version angle were analyzed on 3-month standardized radiographs postoperatively. The displacement criteria for COR (3 mm superior and 5 mm medial) suggested by Dastane et al [19] were applied to both groups. Additionally, the position of center of the prosthetic hip was categorized according to the four-zone system described by Pagnano el al [2] in order to assess the initial position of the acetabular cup (1 = inferomedial, 2 = superomedial, 3 = superolateral, and 4 = inferolateral). The postoperative LLD (as absolute values) was assessed using the method of Ranawat el al [20]. The actual values for each postoperative measure were also obtained using the size of the prosthetic head implanted (Fig 1B). Radiographic cup inclination was defined as the angle between the inter-teardrop line and the long axis of the ellipse (Fig 1B). We used the method described by Lewinneck [9,21] for the measurement of anteversion angle. The serial radiographs were analyzed regarding loosening of each component [22,23] and dislocation.

We compared the perioperative changes in the COR and offset, LLD, and radiographic cup orientation between the two groups. To evaluate whether a learning curve exists for the optimal cup positioning with respect to COR position and orientation, we also compared the results of the early (before 50 THAs) and the late cases (after 50 THAs) within a consecutive series of the less-experienced surgeon based on previous findings that surgeons reached competence in cup orientation within 50 THA cases [24]. When measuring the change in COR, a positive value implies a superior or medial displacement of the COR, and a negative value implies an inferior or lateral displacement of the COR compared to the native COR. Two independent observers who were not involved in any of the operations performed all radiographic measurements. One observer (one of the authors) repeated the measurement after an interval of 1 month to assess intra-observer reproducibility. All radiographs were presented in a random sequence to ensure that the observers were blind to the surgeon’s identity.

### Statistical analysis

Statistical analysis was performed using SPSS (ver. 21; SPSS, Chicago, IL, USA). The intraclass correlation coefficient (ICC) with a 95% confidence interval (CI) was used to assess intra- and inter-observer reliability for each measurement. Independent *t*-tests were performed to compare the groups in terms of age, BMI, cup size, value of the vertical and horizontal displacement of the COR, offset, LLD, and the cup profiles involving inclination and version angle. A chi-squared test or Fisher’s exact test was used to compare the groups with regard to sex distribution, initial cup position, the number of cups within each safe zone, cup outliers, and the dislocation rate. An odds ratio (OR) and related *p*-value were each reported for cups placed outside each safe zone. Changes between the pre- and postoperative COR in each group were analyzed using a paired *t*-test. All measurements are expressed as means ± standard deviations. Statistical significance was set at *p*<0.05 for all analyses.

## Results

**Fig 2:**
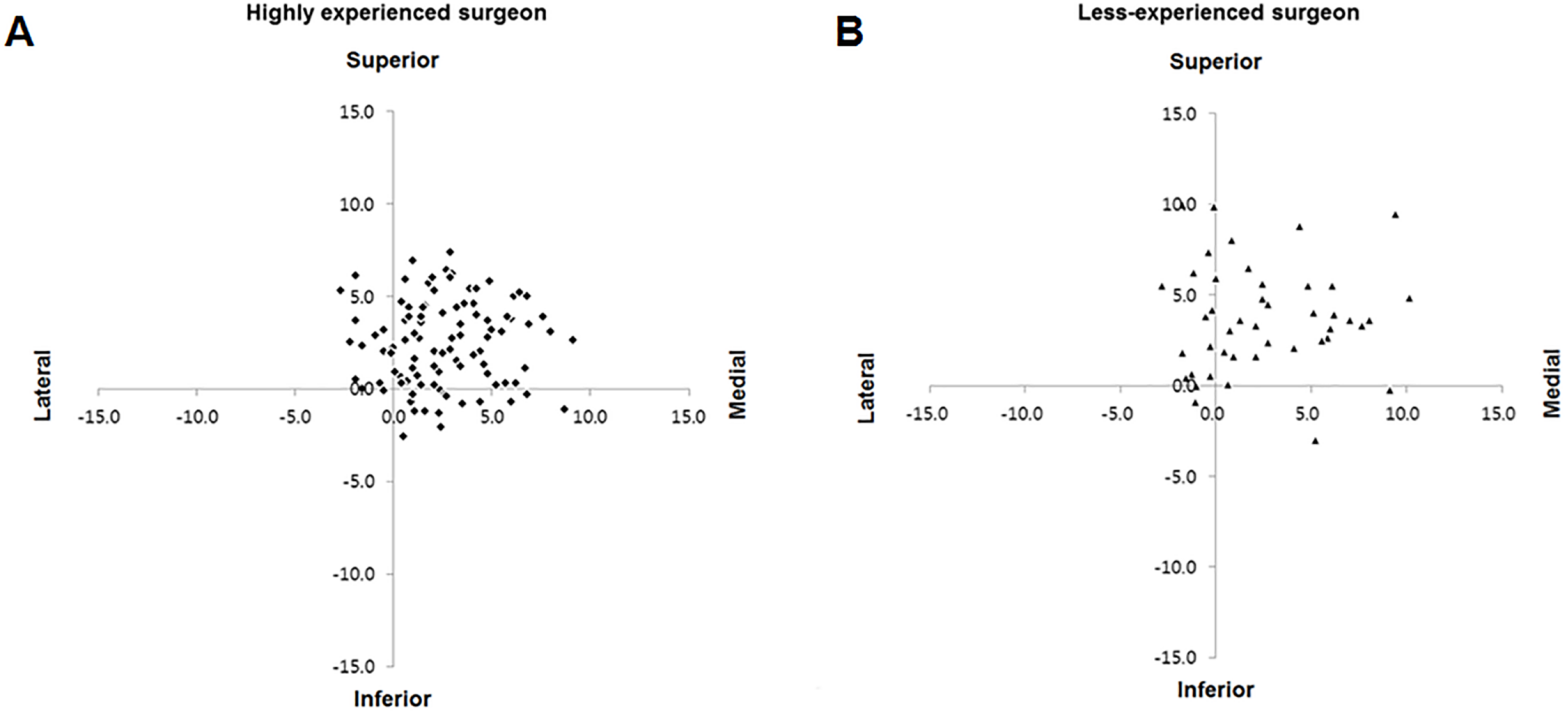
Scatter plots for the perioperative change of COR. Scatter plots of postoperative COR of (A) the highly experienced surgeon, and (B) the less-experienced surgeon are shown relative to the native hip center (0, 0).

**Fig 3:**
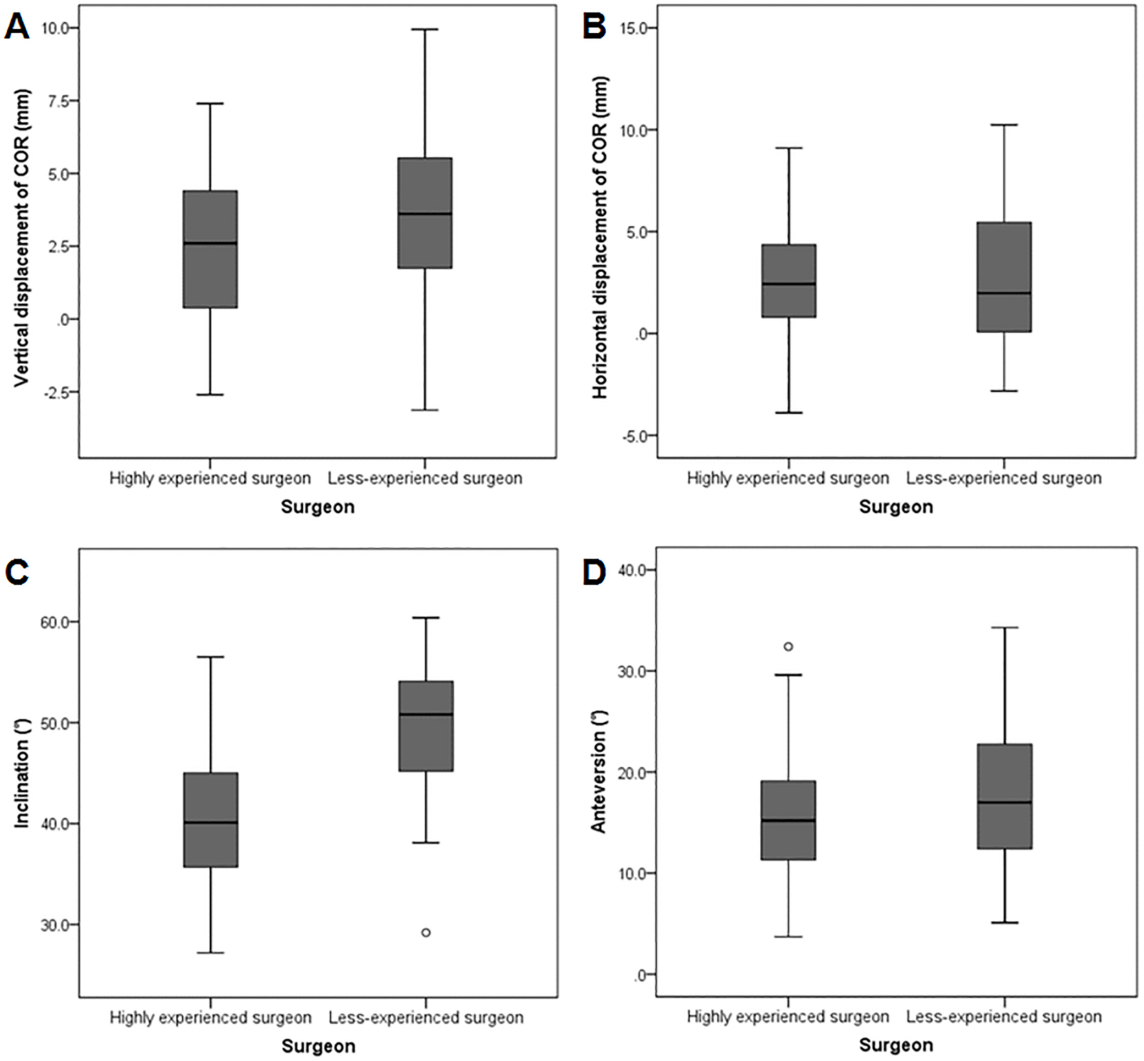
Clustered box plots for each hip parameter following THA. Clustered box plots of (A) the vertical displacement of the COR, (B) the horizontal displacement of the COR, (C) the inclination of the acetabular component, and (D) the anteversion of the acetabular component for two surgeons with different levels of surgical experience.

**Fig 4:**
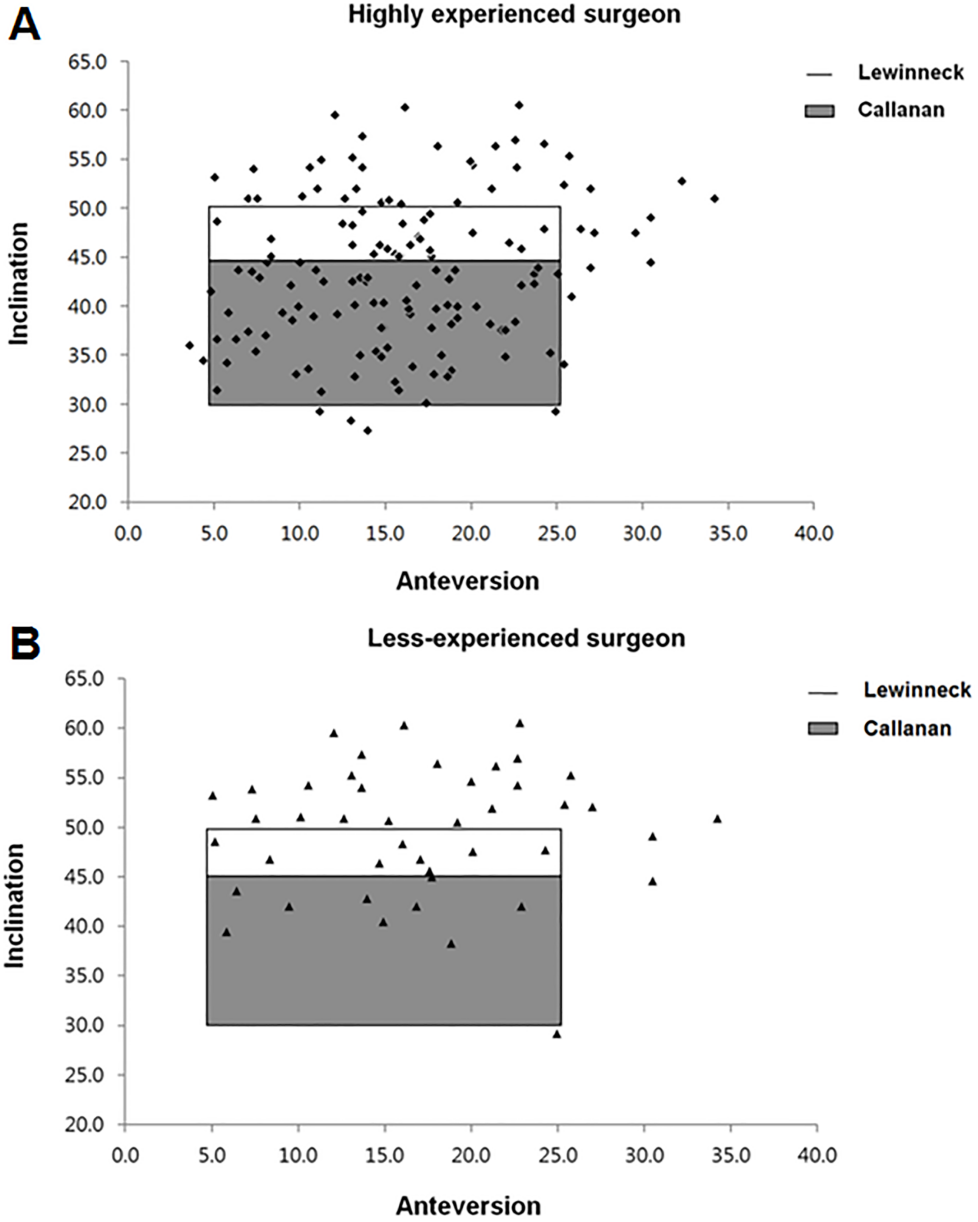
Scatter plots for acetabular cup positioning. Scatter plots of cups placed by (A) the highly experienced surgeon, and (B) the less-experienced surgeon based on the safe zones of Lewinnek et al and Callanan et al.

Perfect intra-observer reproducibility and inter-observer variability were demonstrated for all parameters (Table 2). Cups placed by the highly experienced surgeon showed a smaller perioperative variation in COR position, as well as more accurate and consistent cup positioning (Figs 2 and 3). In addition, the cups were more likely to be located within both safe zones compared to those positioned by the less-experienced surgeon (Fig 4).

**Table 2:** ICC* values for intra- and inter-observer reliability for each measurement.

Parameter	Intra-observer (R) (95% CI)	Inter-observer (R) (95% CI)
Vertical height of COR	0.95 (0.92 to 0.97)	0.92 (0.88 to 0.94)
Horizontal distance of COR	0.94 (0.90 to 0.96)	0.90 (0.85 to 0.93)
Cup inclination angle	0.95 (0.91 to 0.98)	0.93 (0.88 to 0.96)
Cup anteversion angle	0.97 (0.95 to 0.99)	0.95 (0.93 to 0.97)

* ICC = Intraclass Correlation Coefficient

COR = center of rotation

CI = confidence interval

The results for change in the COR in the two groups are summarized in Table 3. The COR was significantly displaced in both groups in the superior and medial directions postoperatively compared with the preoperative native COR (*p*<0.001, for the superior direction; *p*<0.001, for the medial direction) (Fig 2). The mean vertical displacement of the COR was significantly lower in the group operated on by the highly experienced surgeon (*p* = 0.009); however, no significant difference was observed in the mean horizontal displacement of the COR between the two groups (*p* = 0.870) (Table 3). Given that the cut-off values were 3 mm in the superior direction and 5 mm in the medial direction, a significantly higher percentage of hips that had a vertical displacement < 3 mm (superior) was found in the highly experienced surgeon group (58% vs. 39%, *p* = 0.037). A significant difference was also found in the number of hips with a horizontal displacement < 5 mm (medial) (83% vs. 68%, *p* = 0.043). There was a significant difference between the groups in the initial cup position based on the four-zone system (*p* = 0.027); most of the cups were placed within zone 1, and no cups were within zone 2 or 3 in both groups (Table 3).

**Table 3:** Comparison of hip center of rotation and offset following THA according to type of surgeon.

Parameter	Highly experienced surgeon	Less-experienced surgeon	*p*-value
Comparison between pre- and post-operative COR (mm; mean ± SD; 95% CI)			
Vertical displacement of COR	2.5 ± 2.3 (2.0 to 2.9)	3.7 ± 2.9 (2.8 to 4.6)	**<0.001**^**†**^
Horizontal displacement of COR	2.6 ± 2.6 (2.1 to 3.1)	2.7 ± 3.4 (1.7 to 3.7)	**<0.001**^**†**^
Comparison between the groups (mm; mean ± SD; range)			
Vertical displacement of COR	2.5 ± 2.3 (-2.6 to 7.4)	3.7 ± 2.9 (-3.1 to 10.0)	**0.009**^#^
Horizontal displacement of COR	2.6 ± 2.6 (-2.7 to 9.1)	2.7 ± 3.4 (-2.8 to 10.2)	0.870^#^
Acetabular offset (mm; mean ± SD; range)			
Preoperative	30.2 ± 2.8 (23.4 to 39.7)	30.2 ± 2.7 (25.3 to 35.0)	0.876^#^
Postoperative	27.7 ± 2.9 (21.0 to 35.1)	27.5 ± 4.1 (18.5 to 35.6)	0.757^#^
Femoral offset (mm; mean ± SD; range)			
Preoperative	37.7 ± 4.3 (29.0 to 48.8)	38.3 ± 4.3 (31.4 to 52.5)	0.449^#^
Postoperative	39.3 ± 4.1 (32.6 to 49.9)	39.6 ± 3.5 (33.1 to 47.6)	0.633^#^
Hip offset (mm; mean ± SD; range)			
Preoperative	68.0 ± 5.3 (56.1 to 81.8)	68.5 ± 5.6 (57.8 to 87.2)	0.606^#^
Postoperative	67.0 ± 5.6 (53.6 to 79.3)	67.1 ± 5.8 (55.3 to 79.0)	0.902^#^
Zone (n; %)			**0.027**^**‡**^
1: inferomedial	99 (98)	39 (89)	
2: superomedial	0 (0)	0 (0)	
3: superolateral	0 (0)	0 (0)	
4: inferolateral	2 (2)	5 (11)	

# Independent *t*-test

† Paired *t*-test

‡ Fisher’s exact test

COR = center of rotation

SD = standard deviation

CI = confidence interval

Hip offset = Acetabular offset + Femoral offset

Comparisons of the preoperative and postoperative acetabular offset, femoral offset and hip offset between groups showed no statistically significant differences (Table 3).

The mean postoperative LLD was lower in the highly experienced surgeon group (2.3 ± 1.9 mm; range, 0.1 to 7.4) compared with the less-experienced surgeon group (4.9 ± 3.6 mm; range, 0.3 to 17.1, *p*<0.001). 57 patients (56%) in the highly experienced surgeon group had a shorter limb postoperatively (operated limb 0.1 to 7.4 mm shorter than the contralateral side, mean 2.6 mm). Whereas, 33 patients (75%) in the less-experienced surgeon group had a shorter limb (operated limb 1.0 to 17.1 mm shorter than the contralateral side, mean 5.4 mm, *p*<0.001).

A significant difference in the mean radiographic inclination angle of the cup was observed between the two groups (*p*<0.001), while no significant difference was observed in the mean radiographic anteversion angle (*p* = 0.194) (Table 4). Significantly more cups were within each safe zone for combined inclination and anteversion in the highly experienced surgeon group (Table 4, Fig 4): 81% vs. 39% for Lewinnek (*p*<0.001), and 71% vs. 20% for Callanan (*p*<0.001), respectively. The number of cups within the safe zone for inclination only was also determined to be significantly higher for the highly experienced surgeon; however, no significant difference was identified in the number of cups within the safe zone for anteversion only (Table 4). The number of cup inclination outliers was significantly higher for the less-experienced surgeon (OR = 11.97, 95% CI = 4.95 to 29.0, *p*<0.001 for Lewinnek; OR = 11.53, 95% CI = 4.96 to 26.83, *p*<0.001 for Callanan, respectively). However, there was no significant difference in the outliers of cup anteversion (OR = 1.72, 95% CI = 0.61 to 4.87, *p* = 0.301).

**Table 4:** Comparison of accuracy in acetabular cup placement.

Parameter	Highly experienced surgeon	Less-experienced surgeon	*p*-value
Cup inclination (°; mean ± SD; range)	40.7 ± 6.4 (27 to 56)	49.4 ± 6.5 (29 to 60)	**<0.001**^#^
Cup anteversion (°; mean ± SD; range)	15.7 ± 6.1 (4 to 32)	17.2 ± 7.2 (5 to 35)	0.194^#^
Cups within safe zones for combined (n; %)			
Lewinneck	82 (81)	17 (39)	**<0.001**^†^
Callanan	72 (71)	9 (20)	**<0.001**^†^
Cups within safe zones for inclination (n; %)			
Lewinneck (safe range, 30° to 50°)	92 (90)	19 (43)	**<0.001**^†^
Callanan (safe range, 30° to 45°)	79 (78)	10 (23)	**<0.001**^†^
Cups within safe zones for anteversion (n; %)			
Lewinneck, or Callanan (safe range, 5° to 25°)	92 (90)	37 (84)	0.301^†^

# Independent *t*-test

† Chi-square test

SD = standard deviation

There were no statistically significant differences between the early (15 hips) and late series (29 hips) of the less-experienced surgeon in both the COR displacement (mean vertical displacement, 4.5 ± 3.6 mm vs. 3.2 ± 2.5 mm, *p* = 0.186; mean horizontal displacement, 3.4 ± 3.4 mm vs. 2.5 ± 3.3 mm, *p* = 0.401) and the cup orientation (mean inclination, 49.0 ± 8.6° vs. 49.7 ± 5.3°, *p* = 0.758; mean anteversion, 19.1 ± 7.3° vs. 16.2 ± 7.1°, *p* = 0.217). Although the difference was not significant, the superior displacement of the COR was found to decrease in his late cases of after 50 THAs: 13 (45%) of the 29 hips in his late cases had a vertical displacement < 3 mm (superior) compared to only 4 (27%) of the 15 hips in his early cases.

Comparison of mean preoperative and postoperative HHS revealed no differences between the two groups. The mean preoperative HHS was 45.3 ± 5.7 points for the highly experienced surgeon group and 46.7 ± 6.2 points for the less-experienced surgeon group (*p* = 0.338). This improved 94.1 ± 5.0 points and 92.9 ± 7.1 points at the latest follow-up, respectively (*p* = 0.167). At the time of the latest follow-up, there had been no loosening of the acetabular and femoral components in both groups, and no patient in each group underwent revision surgery due to aseptic loosening. Three hips (7%) dislocated in the less-experienced surgeon group at a mean follow-up of 39 months (range, 24 to 84), compared to none (0%) in the expert group (*p* = 0.027).

## Discussion

The position and alignment of the acetabular component, which has a direct effect on the outcome of THA, is critical in ensuring the stability of the prosthetic hip and the longevity of the prostheses [10]. Factors related to satisfactory cup placement include the surgical approach, body mass index, available methods to assist surgeons in placing cups, and surgical experience. We expect that the more operations we perform, the more accurate our results. The questions arising from this study are whether implantation of an acetabular component in an anatomical position provides a native COR in cementless primary THA, and whether a THA performed by a surgeon with a high level of surgical experience results in a smaller variation in COR position postoperatively and a lower incidence of cups positioned outside the safe zones compared to a surgeon with a low level of experience.

The precise restoration of hip biomechanics is one of the most important goals of THA. Several studies have demonstrated the importance of the reconstructed COR [19]. Wan et al [7] compared the position of the COR of an operated hip with a normal hip and found that the mean vertical and horizontal changes in the COR were 5.1 ± 4.5 mm (superior) and 4.4 ± 4.4 mm (medial), respectively; among 49 hips, 5 (10.2%) had a COR change greater than 13 mm in the superior direction, and 13 (26.5%) had a COR change greater than 7.5 mm in the medial direction. A superior displacement of the COR, in particular, can result in shortening of the leg, bony impingement, decreased abductor muscle tension, and loosening of the implants. Using a computer model, Kurtz et al [25] validated the finding that as the variance of the COR increases, an increased offset stem is required to avoid impingement and restore the femoral offset. However, increasing the offset by more than 5 mm from the normal hip can lead to increased bearing-surface wear [26]. Accordingly, Dastane et al [19] suggested that displacement criteria of 3 mm superior and 5 mm medial be used as the safe range for COR change. In particular, to maximize muscle function and minimize wear, the superior displacement of COR must be kept within 3 mm of normal. These findings are consistent with our data, in which the hip COR was significantly displaced in the superior and medial directions from the native COR in both groups due to the reaming of the acetabulum and the medialized cup placement. However, the 95% CIs differed significantly between the two groups with respect to the vertical displacement of the COR (*p* = 0.009) (Table 3); most of the values within the 95% CI of the less-experienced surgeon were more than 3 mm superior (95% CI, 2.8 to 4.6), and all of the three dislocated hips in this group had a superior displacement more than 3 mm (4.7 mm, 5.3 mm, and 9.4 mm, respectively).

Superior or lateral placement of the cup has been described as a risk factor for aseptic loosening of the implants because joint loads are greater in the superior-lateral position than in the inferior-medial position. Johnston et el [27] demonstrated that loads are minimized by placing the hip center as inferiorly, medially, and anteriorly as possible with their mathematical analysis model. Furthermore, Pagnano et al [2] found that an anatomical hip center was associated with significantly lower loosening and aseptic revision rates for both acetabular and femoral components. In our study, significantly more cups were placed within zone 1 (inferomedial) by the highly experienced surgeon compared to the less-experienced surgeon, which did not lead to a difference in loosening of the acetabular or femoral component, or subsequent revision at a minimum 2-year follow-up.

Little et al [26] demonstrated that an acetabular component abduction of 45° or greater was associated with a 50% increase in linear wear and a 44% increase in volumetric wear per year compared with an abduction angle of less than 45°. Similarly, Hirakawa et al [28] suggested that an increase of more than 45° in the abduction angle leads to an increase in contact stress and subsequent mechanical failure. Malposition of the acetabular component and soft tissue imbalance are also thought to be main causes of dislocation [8,12]. In the present study, the mean inclination angle was greater than 45° in the group operated on by the less-experienced surgeon but not in the group operated on by the highly experienced surgeon (40.7° vs. 49.4°, *p*<0.001). Three hips (7%) subsequently dislocated, all in the former group. We cannot explain this by a single factor because the cause of dislocation is multifactorial. However, our results are consistent with previous findings that the main reason for the higher rate of dislocation of inexperienced surgeons is malpositioning of the cup [8].

Studies related to the use of freehand cup positioning have reported inconsistent acceptable angle ranges for optimally positioned cups and the proportion of cups within safe zones in primary THA. According to these studies, the percentage of hips located within the safe zones varies from 70.5% to 25.7%. Callanan et al [10] evaluated the acetabular cup positioning performed by several experienced surgeons, and then reported the percentage of cups located within their modified safe zone: 62% for inclination, 79% for anteversion, and 47% for combined inclination and anteversion. Bosker et al [29] reported the percentage of acceptably placed cups using the Lewinnek safe zone to be 85.2% for inclination, 82.7% for anteversion, and 70.5% for combined. Saxler et al [30] reported 25.7% of cups to be within the combined Lewinnek safe zone, which was lower than in the other studies. We found that the number of cups within each safe zone for inclination only, or for combined inclination and anteversion was significantly higher for the highly experienced surgeon (Table 4). However, no significant difference was determined for anteversion only (*p* = 0.301). Our findings are consistent with a previous study in which they suggest a lower-volume surgeon’s greater risk of cup malpositioning is due to a lack of accuracy in cup abduction, not cup version [10].

Reize et al [31] recently reported that there was no significant difference in acetabular cup orientation when the level of surgical experience was taken into account; thus, surgical experience did not influence the accuracy of acetabular cup positioning. Unlike our study, they divided a total of 78 THAs into two groups of surgeons (a group of five relatively experienced surgeons, and a second group of seven relatively inexperienced surgeons); therefore, a small number of THAs (~ 3 to 13) were performed per surgeon. In contrast, the present study determined significant differences in the radiographic and clinical outcomes between surgeons with different levels of surgical expertise, such as change in the COR, initial cup position, LLD, cup orientation, number of cups within safe zones, and dislocation rate. To improve the accuracy and consistency of implantation of the acetabular component for a novice surgeon, the use of an alignment guide should be considered for cup orientation within the safe zones, and in particular for the inclination.

There were several limitations to this study. First, the study population was not large enough to comment a difference in the frequency of an uncommon event like dislocation. In particular, the group operated on by the less-experienced surgeon had a relatively small cohort of patients, which, however, has to be allowed to maintain a difference in surgical experience as a critical variable in the study. Second, the duration of follow-up was short with a minimum follow-up of 2 years and an average of 3.3 years. Longer-term follow-up will answer the question about whether a more superiorly displaced hip center would lead to adverse clinical outcomes more frequently such as loosening of acetabular and/or femoral component. Third, the method used in the current study to assess the COR position cannot be applied to hips with an abnormal anatomy of the acetabulum, such as dysplastic hips. Lastly, the radiographic outcomes of only two surgeons were evaluated. Furthermore, we were not able to confirm significant improvements in cup placement with an increase in the number of operations performed. We suggest that further research is required to determine this. Our results are, however, strengthened by the fact that we had no loss to follow-up postoperatively. In addition, the results of this study may help experienced as well as inexperienced surgeons equalize leg-length and restore offset in primary THA, since those important factors are directly influenced by a combination of COR position and femoral component.

This study has shown that the postoperative COR tends to be displaced in the superior and medial directions from the native COR after cementless primary THA. Nevertheless, our findings suggest that an increase in surgical experience can strongly improve both the accuracy and consistency of acetabular cup placement in terms of COR change and cup inclination. At short-term follow-up, however, there was no difference in clinical outcomes with regard to component loosening, revision rates, and HHS between the two groups except dislocation rates. Longer follow-up and larger studies are required to verify these conclusions.

## Supporting information

S1 File Minimal dataset of this study. (XLS)
